# Important Elements of Quality Home End-of-Life Care in China

**DOI:** 10.1001/jamanetworkopen.2025.31176

**Published:** 2025-09-10

**Authors:** Yinshi Kan, Yao Xiao, Zhaoyu Li, Shan Zhang, Xiaotian Zhang, Xiaodong Wang, Dandan Huang, Minghui Wang, Li Gao, Shuang Gao, Guijun Lu, Xinyan Zhang, Peng Yue

**Affiliations:** 1 School of Nursing, Capital Medical University, Beijing, China; 2 Department of Nursing, Peking University Third Hospital, Beijing, China; 3 Peking University Shougang Hospital, Beijing, China; 4 Puhuangyu Community Health Service Center, Beijing, China; 5 Beijing Luhe Hospital, Beijing, China; 6 Beijing Tsinghua Changgung Hospital, Beijing, China

## Abstract

**Importance:**

The efficacy of home end-of-life care in enhancing the quality of life for terminally ill patients and families has been well documented. While previous studies have explored perspectives on quality home palliative care and end-of-life care in several countries, limited knowledge exists regarding its specific components in the Chinese context.

**Objective:**

To explore the core elements that constitute quality home end-of-life care in China.

**Design, Setting, and Participants:**

This qualitative study integrated semistructured interviews, participant observation, and document and record collection. Data were collected from March to November 2024 in 4 health care organizations with hospice wards in Beijing. Purposive sampling was used to recruit terminally ill patients and families who had received home end-of-life care, as well as professionals who had provided care. All data were analyzed using thematic analysis.

**Main Outcomes and Measures:**

Core elements of quality home end-of-life care in China.

**Results:**

A total of 39 participants were included: 12 patients (5 [41.7%] female and 7 [58.3%] male), 4 of whom were interviewed (mean [SD] age, 74 [3.5] years); 13 family members (6 [46.2%] female and 7 [53.8%] male; 6 interviewed, primarily spouses [mean (SD) age, 69 (6) years] and adult children [mean (SD) age, 41 (5.3) years]); and 14 professionals (mean [SD] age, 42 [7.8] years; 11 [78.6%] female and 3 [21.4%] male). Observations and interviews were conducted mainly during 19 home visits. Four themes and 10 subthemes were identified: (1) preliminary basis for home-based services, (2) communication strategies and practical support, (3) long-term stable care relationships, and (4) fulfilling end-of-life preferences in practice. These elements reflected a dynamic progression of trust relationships between patients, families, and professionals. While some elements were represented in previous studies, we found some unique aspects related to mutual efforts on both sides of the service and professionals’ approach to the inconsistent needs of patients and families.

**Conclusions and Relevance:**

In this qualitative study, quality home end-of-life care in China can be described as a dynamic process of building and maintaining trust relationships between professionals, patients, and families. The findings provide a distinct understanding and may serve as a reference for developing a quality assessment framework for home end-of-life care. Future research should further develop this framework and evaluate effective strategies for delivering quality home end-of-life care within distinctive cultural and policy contexts.

## Key Points

**Question:**

What are the important elements of quality home end-of-life care in China?

**Findings:**

In this qualitative study of 39 participants, 4 key elements of quality home end-of-life care in China were identified: preliminary basis for home-based services, communication strategies and practical support, long-term stable care relationships, and fulfilling end-of-life preferences in practice. These 4 constituent elements can be linked to a dynamic progression of trust relationships.

**Meaning:**

These 4 elements can serve as a practical reference for policymakers to develop quality assessment and improvement systems for home end-of-life care within the distinctive cultural context of China.

## Teaser

This qualitative study surveyed terminally ill patients, their families, and professional caregivers to identify key elements of quality home end-of-life care in China.

## Introduction

Global population aging has led to increased mortality.^1^ Widespread promotion of palliative care and end-of-life care has emerged as a critical health care strategy.^2^ As most terminally ill patients tend to spend the last stages of their lives at home, the benefits of home end-of-life care for patients and families have been widely reported.^3,4,5^ However, discrepancies exist between current clinical practices and ideal standards due to varying policy support and service models globally.^6^

Previous studies have identified several critical components of successful home palliative care and end-of-life care, including dealing with complex symptoms, ensuring a sense of security in the home, respecting individuality, being proactive, and providing clear and rapid service.^7,8^ These components offer a valuable framework, but their practical implementation requires cultural adaptability and sensitivity to local norms, family dynamics, and health system contexts.

In China, home end-of-life care services operate through 3 models: (1) a hospital-led model, which mainly includes telehealth for homebound patients and home visits for discharged patients; (2) a community-based model, primarily embedded in the family doctor contracting service, where professionals conduct home visits after assessing the patient’s home condition; and (3) hospice institutions (eg, set up by the Li Ka Shing Foundation), which help underprivileged people with advanced cancer to obtain basic painkillers and provide basic life care.^9,10,11^ Furthermore, home end-of-life care in China is shaped by culture and systemic factors, including filial piety, family-dominated medical decision-making, and the general avoidance of discussing death, alongside structural challenges, including fragmented service delivery, a shortage of human resources, and limited public awareness.^12,13,14^ These contextual characteristics may influence China’s perspectives on the quality of home end-of-life care.

Accordingly, this study aimed to explore the core elements that define quality home end-of-life care in China, based on the experiences and feedback of relevant stakeholders. It further examines similarities and differences in home end-of-life care priorities in global contexts.

## Methods

### Study Design and Participants

This qualitative study followed the Standards for Reporting Qualitative Research (SRQR) reporting guideline.^15^ This study integrated participant observation, semistructured interviews, and document and record collection from March to November 2024 in 4 health care institutions with distinct hospice care model wards in Beijing.^16^ Participants were purposively sampled and included terminally ill patients (advanced cancer or multiple noncancer comorbidities, survival expectancy <6 months as assessed by 2 physicians) and families (cared for patients for ≥3 months) who had received home end-of-life care services, as well as professionals who had provided the services. Professionals conducted home end-of-life care mainly through home visits, internet clinics, and online communication after discharge from the hospital. Sampling was done for each role on the team to ensure adequate representation.

### Data Collection

The researchers collaborated with hospice ward directors from 4 institutions and observed 4 home hospice teams’ professional-patient-family interactions and service evaluations. The researchers stopped observations and recordings if patients or families declined, in order to respect participants’ autonomy. The field notes, including service time, location, participants, key dialogues, and stakeholder perspectives were promptly documented after observation. Documents, including photographs, family meeting transcripts, memory albums, and WeChat conversations reflecting service quality, were also collected. Reflective journals recorded researchers’ observational impressions for minimizing potential biases. Researchers developed 3 semistructured interview outlines for patients, families, and professionals (eTable in Supplement 1). Face-to-face interviews were conducted at the later stages of the home end-of-life care service, after a trusting relationship had been established. Interviews were family-oriented, as some patients were too frail to be interviewed. Patients and families were interviewed at home, and professionals were interviewed in quiet offices within the wards. Interviews lasted 20 to 70 minutes. Audio recordings were transcribed verbatim. Ethical approval was obtained from the Ethics Committee of Capital Medical University, and written informed consent was obtained from all participants.

### Data Analysis

We conducted thematic analysis following Braun and Clarke’s 6-step framework.^17,18^ All data (verbatim transcripts of interviews, field notes, and care-related documents) were imported into NVivo 14 (QSR) for storage and analysis. The first author (Y.K.) familiarized themself with the data, conducted initial open coding, and generated preliminary themes. The entire research team then engaged in iterative discussions to refine and define themes, attending to both semantic and latent content. Weekly team discussions, involving researchers with hospice care backgrounds and qualitative research experience, resolved discrepancies until consensus was achieved. Similarities and differences across patients, families, and professionals were analyzed to ensure a rich, multiperspective understanding of home end-of-life care quality elements in China.

## Results

A total of 39 participants (12 patients [5 (41.7%) female and 7 (58.3%) male], 13 family members [6 (46.2%) female and 7 (53.8%) male], and 14 professionals [11 (78.6%) female and 3 (21.4%) male]) were included. Fifteen participants were only observed, while 24 were both observed and interviewed. Among the interviewed subgroup, 4 were patients (mean [SD] age, 74 [4] years; 3 of whom have since died), 6 were family members (primarily spouses and adult children [mean (SD) age, 69 (6) years and 41 (5) years, respectively]), and 14 were professionals (mean [SD] age, 42 [8] years). These sessions constituted a total of 19 home visits. The professional staff included diverse roles essential to comprehensive home end-of-life care, including doctors, nurses, social workers, a case manager, general practitioners, and volunteers (Table 1). The thematic analysis yielded 4 themes and 10 subthemes (Table 2).

**Table 1.** Detailed Characteristics of Patients, Families, and Professionals

Participants	No. (%)		
	Patients (n = 12)	Family members (n = 13)	Professionals (n = 14)
Sex			
Female	5 (41.7)	6 (46.2)	11 (78.6)
Male	7 (58.3)	7 (53.8)	3 (21.4)
Data collection			
Observation only	8 (66.7)	7 (53.8)	0
Observation + interview	4 (33.3)	6 (46.2)	14 (100)
Age, mean (SD), y^a^	74 (4)	Spouse: 69 (6); child: 41 (5)	42 (8)
Main diagnosis^a^			
Pancreatic cancer	2 (50.0)	NA	NA
Breast cancer	1 (25.0)	NA	NA
No cancer with multimorbidity	1 (25.0)	NA	NA
Family status^a^			
Married	1 (25.0)	6 (100)	NA
Widowed	3 (75.0)	0	NA
Relationship to patient^a^			
Spouse	NA	3 (50.0)	NA
Child	NA	3 (50.0)	NA
Frequency of contact to patient^a^			
Every day	NA	5 (83.3)	NA
3-6 d/wk	NA	1 (16.7)	NA
Profession			
EOLC doctor	NA	NA	2 (14.3)
EOLC nurse	NA	NA	3 (21.4)
EOLC social worker	NA	NA	4 (28.6)
EOLC case manager	NA	NA	1 (7.1)
General practitioner	NA	NA	2 (14.3)
EOLC volunteer^b^	NA	NA	2 (14.3)
Time in practice, mean (SD), y			
Work experience	NA	NA	11 (7)
Experience in EOLC	NA	NA	3 (4)
Practice location			
Urban	NA	NA	11 (75.9)
Suburbia or rural	NA	NA	3 (24.1)
Institutional level			
First level	NA	NA	4 (28.6)
Second level	NA	NA	3 (21.4)
Third level	NA	NA	7 (50.0)

Abbreviations: EOLC, end-of-life care; NA, not applicable.

^a^ Only reported for the patients and family members who were interviewed.

^b^ One volunteer’s background is in nursing, while another’s is in Chinese medicine, so they are classified as professionals, but they do not offer services related to their professional backgrounds during their service. Instead, they provide comfort care, such as aroma pampering services.

**Table 2.** Summary of the Themes, Subthemes, and Codes

Themes and subthemes	Supporting quotations
**Preliminary basis for home-based services**	
Comprehensive home condition assessment	
Patient’s home status	“Some patients live in particularly cluttered and noisy home environments. This is not appropriate.” (N1, female, interview) “Professionals frequently ask families, ‘Is there an air mattress at home, or a home-type oxygen machine...’” (Observation) “I instructed him to take medication, which helped control his gastrointestinal symptoms for him to remain at home. This assisted me in assessing his suitability for in-home services.” (D1, female, interview)
Degree of family support	“If it is mainly the nanny to take care of [the patient], the degree of support that can be received is definitely not the same as if the family to take care of [the patient].” (GP1, female, interview) “Some family members are so uncooperative that it is difficult to communicate effectively.” (Observation)
Comprehensive judgment on whether to intervene	“Preassessment is central, and there are many factors that need to be taken into account to determine whether home end-of-life care can be achieved.” (D2, male, interview) “Although [the patient’s] family support was weak, his own cooperation was high, his symptoms were still mild, and his basic needs were clearly assessed, and I felt could provide him with support.” (GP1, female, interview)
Trust-building as access gateway	
Initial wariness	“I’d like you to come to my home..., but it’s messy....It’s better not to have too many people.” (Observation) “You’re coming to my home....I’m afraid I’ll hold you up.” (Observation)
Feeling genuine and caring	“Initially, I have been observing you (behavior), then I can feel that your team really wanted to help me.” (F6, female, observation) “We can only enter (the home) if the patient and family trust us.” (SW1, female, interview)
**Communication strategies and practical support**	
Easy-to-understand communication	
Patient listening and company	“There are many things she (patient) does not want to say to me, probably because she doesn’t want to add to my burden....With your company and listening, my mother was able to express the true feelings of her heart.” (F4, male, interview) “Your company has decreased my pain by one-third.” (P3 said to V2, observation) “Your son is very caring and you can rest assured that we will always help you too.” (Observation)
Using understandable and acceptable words	GP2 when prescribing pain medication: “You don’t sleep well. This medicine will reduce your pain and also improve your sleep, you can feel more comfortable.” Additionally, when explaining the use of medicines, he would write simplified instructions on paper to enhance understanding and recall. (GP2, male, observation) “Patients and families have different levels of openness (about death), and you need to know how to measure your words.” (V4, female, interview)
Practical professional support	
Basic symptom management	“A physician’s mastery of the use of analgesic medications for home patients is critical.” (GP2, male, interview) “I took a video of you (Chinese medicine volunteer) massaging him (patient). I massaged my husband according to your method and he said he was much more comfortable. Your method is very useful. It was comforting to see that he could feel better physically.” (F2 said to V1, observation)
Progressive psychological support	“Initially, she and her husband were not talkative, after I used dignity therapy on them, they were willing to express themselves happily. I think it was tapping into the spiritual part of life, which was very meaningful.” (SW2, male, interview) “Your sandbox game let him (patient’s young grandson) know that his grandfather was dying and he went back and cried...” (Observation) P2 wrote in the time album: “Thank you for your willingness to listen to my story, your care has left a ray of warm sunshine in my heart.” (Documents and records)
Providing useful information	“I was told in detail what to do (procedures after death)...so that I wouldn’t get very flustered.” (F5, male, interview) “As a convenience, we try to provide them (patients and families) with information about hospitals close to home and what specific services are available [at the hospital].” (CM1, female, interview) “I don’t live with my father (patient), and I would have more peace of mind with in-home remote monitoring.” (Observation)
**Long-term stable relationships**	
Tailored care management	
Establishing home-care WeChat group for each family	“Every family’s situation is different. I can contact you (professionals) directly in the [WeChat] group for any needs. This group is created just for our family and can take into account my individual needs.” (F2, female, interview) “I can be informed of the services you (professionals) are doing for my father through [WeChat] group messages.” (F1, male, interview)
Providing individualized services	“Some patients believe in Buddhism, so that they may need some special services.” (SW3, female, interview) “If the service you give is not what the other person needs, it can add some trouble instead.” (CM1, female, interview) “The services you provide were not cookie-cutter and process-oriented, they are given based on the characteristics of our home.” (F2, female, interview)
Regular case seminars	“Our team discussed and reflected together: Are the patients satisfied? Are the families satisfied? Is the teamwork satisfactory? Are you satisfied with your work?” (D2, male, interview) “Every month, the hospice director of Peking Union Medical College Hospital comes to our department to guide us on improving our cases.” (N3, female, interview)
Flexible home visits mechanism by multidisciplinary teams	
Relatively fixed care roles	“In China, volunteer teams are huge and closely associated with the grassroots level of society. Its power should be put to good use.” (D2, male, interview) “The social worker and volunteers gave us a lot of basic support. In fact, there was no way I could have bathed him without you coming in.” (F3, female, observation)
Dynamic needs-based roles	“When the social worker gives feedback that the patient’s symptoms of dyspnea are getting worse, the doctor and I will make a timely home visit to address it.” (D2, female, interview) “There aren’t that many doctors and nurses to do home services every time.” (GP1, female, interview)
Reciprocal relationship between professionals and families	
Feedback from life experience	“I act as a family coordinator at home. If I have to communicate something, I will analyze the pros and cons of the matter from my son’s and my husband’s perspectives. It’s the same with your work, make sure you communicate from the other person’s point of view.” (F2, female, observation)
Taking one’s story advocacy	“My mom has been a teacher all her life, she didn’t expect to end up contributing to this (anonymous advocacy case), she was really very happy.” (F4, male, interview) “More people should be made aware of our services and know that there is still this path to take.” (D1, female, interview) “I really didn’t know about this service before, and to be able to come to my home and support [my father], I’m very grateful and would like to support your work.” (F3, male, observation)
**Fulfilling preferences for end-of-life in practice**	
Providing the final safety net	
Accurately identifying signs of inability to continue living at home	“My father was already incontinent, he felt couldn’t stay at home and had to rush to the hospital.” (F9, male, observation) “Pancreatic cancer progresses very quickly, your father can’t stay at home in his current condition.” (Observation)
Smooth referrals to hospital	“Before I established a relationship with you (professionals), I had no idea what else I could do, after contacting you (professionals), the head said she would definitely leave a hospital bed for me, and I felt like I got a backer in my old age.” (P1, male, interview)
Negotiating the fulfillment to die at home	
Promoting harmonization of family decision-making	“I think the most important thing is to be able to get a unity of opinion from the whole family. We usually have a family meeting to let them know what the patient really wants (to go home) and try not to let the patient have regrets.” (N1, female, interview) “They (families) understood and agreed with my decision to want to go home. I thank you (professionals) all so much.” (Observation)
Recognizing and managing anticipatory symptoms at home	“We will inform the family about the possible risks and countermeasures that may arise when the patient goes home and explain them clearly before letting them go back.” (CM1, female, interview)
Ongoing online support during the dying period	“Some families care for patients at home and get flustered, so they will send you messages at any time and we need to respond to it promptly.” (N3, female, interview) N2 and F9 have been communicating on WeChat; F9 wrote: “Thank you for your help, fulfilling my grandfather’s wish...allowing me to be there for his final days...my grandfather passed away yesterday.” (Documents and records)
Extending care to families after patient’s death	
Grief counseling	Families often express: “I could feel that someone was helping me get through this hard time.” (Observation) One family member said, “I’d like to entrust...[my death] in your hands in the future.” (Observation) Professionals emphasize: “The level of grief of the families can reflect to some extent the quality of our services.” (Observation)

Abbreviations: CM, case manager; D, doctor; F, family member; GP, general practitioner; N, nurse; P, patient; SW, social worker; V, volunteer.

### Theme 1: Preliminary Basis for Home-Based Services

#### Comprehensive Home Condition Assessment

Professionals emphasized that home end-of-life care services were not suitable for all patients, and that making an appropriate decision about whether to provide services was crucial. As doctor 2 reported: “Pre-assessment is central, and there are many factors that need to be taken into account to determine whether home end-of-life care can be achieved.” Professionals noted that factors might include the patient’s physical condition at home, home environment, and the competence and willingness of families or other informal caregivers: “Some patients live in particularly cluttered and noisy home environments. This is not appropriate.”

#### Trust-Building as Access Gateway

Some patients and families were initially hesitant to accept home-based services due to emotional concerns or social discomfort. Some families said to professionals: “You’re coming to my home....I’m afraid I’ll hold you up.” As trust gradually built, they felt the genuineness and caring of the professionals and were willing to open their doors. As one social worker stated: “We can only enter (the home) if the patient and family trust us.”

### Theme 2: Communication Strategies and Practical Support

#### Easy-to-Understand Communication

Most families repeatedly described the patient’s painful experience with the disease and their current sense of helplessness. Professionals always listened patiently and gave advice accompanied by comforting physical gestures. Some professionals personally called patients at home to provide comfort and support measures after listening to the detailed accounts of the family in the clinic: “Your son is very caring and you can rest assured that we will always help you too.” All the patients and families appreciated the attitude of the professionals and felt that they received an emotional release. Patients and families generally appreciated the professionals’ use of plain language and an accessible communication style. This is reflected in professionals consciously adapting their explanations to the cognitive level of patients and families and adapting to their acceptance of death topics, maintaining appropriate sensitivity in their communication.

#### Practical Professional Support

All participants emphasized that home end-of-life care focused on direct and practical assistance. Professionals prioritized symptom control through pain diaries, medication adjustments, and traditional Chinese massage. Psychological and spiritual care was also provided through dignity therapy, sand tray games, and memorial albums, all of which helped patients and families alleviate emotional distress. As one patient wrote in the time album: “Thank you for your willingness to listen to my story, your care has left a ray of warm sunshine in my heart.” Professionals also provide practical and actionable knowledge, including guiding families to nearby hospital services for terminally ill patients and families. Family members also advocated for remote home monitoring to enhance safety, with one stating, “I don’t live with my father (patient), and I would have more peace of mind with in-home remote monitoring.”

### Theme 3: Long-Term Stable Care Relationships

#### Tailored Care Management

Since every family’s situation and needs are different, all participants agreed that providing tailored care services was important. Professionals established WeChat groups for each family, including the patient, relatives, and members of the multidisciplinary care team, to enhance communication and provision of tailored care. In addition, each family was assigned a home care file that documented the entire individualized service process. Finally, the team held timely case seminars where members reflected on what they had done well and identified areas for improvement in providing home end-of-life care to patients and families from their respective roles. All patients and families felt the individualized service provided by the professional team and appreciated it: “The services you provide were not cookie-cutter and process-oriented, they are given based on the characteristics of our home.”

#### Flexible Home Visits Mechanism by Multidisciplinary Teams

There is a significant shortage of professionals engaged in home end-of-life care in China. A flexible home visits mechanism has gradually evolved within the multidisciplinary teams to ensure quality care. Social workers and volunteers are involved more frequently. They typically serve in a relatively fixed role in assisting patients and families with basic care, such as cleaning, medication delivery, and companionship. As one family member noted, “The social worker and volunteers gave us a lot of basic support. In fact, there was no way I could have bathed him without you coming in.” They also update the team on the current status of the patients and families and consult with the team on staffing for the next home visit as needed. Doctors and nurses work in specialized medical roles, but they are not always involved in home visits due to limited resources: “There aren’t that many doctors and nurses to do home services every time.” They usually participated in the initial home visits, and subsequent home visits were conducted when a patient had a clear medical necessity.

#### Reciprocal Relationship Between Professionals and Families

As the relationship of trust between the two parties is steadily built up, some families would offer young professionals ideas and practices in dealing with many of their life experiences, including balancing family, work, and health. Professionals also felt empowered by the sincerity of patients and families. Most patients and families said they were previously unaware of home end-of-life care services. Nevertheless, they acknowledged the dedication of the professionals. “I really didn’t know about this service before, and to be able to come to my home and support (my father), I’m very grateful and would like to support your work.” They were willing to authorize their stories to be used by the agency for anonymous publicity to support other families facing end-of-life situations. A reciprocal relationship exists between patients and families and professionals.

### Theme 4: Fulfilling End-of-Life Preferences in Practice

#### Providing the Final Safety Net

Patients and families expect professionals to accurately identify the risk signs that the patient can no longer remain at home (“Pancreatic cancer progresses very quickly, your father can’t stay at home in his current condition”). After a timely clinical decision, most terminally ill patients in urban areas wanted to be smoothly referred to a hospice ward. Even when hospice beds were scarce, teams often reserved one in advance to ensure a seamless transition. This brought great reassurance to families: “Before contacting you, I didn’t know what to do. The head said she’d leave a bed for me, and I felt like I had a backer in my old age.” Such proactive support serves as a crucial safety net at the end of life.

#### Negotiating the Fulfillment to Die at Home

Most terminally ill patients in rural and out-of-town areas, influenced by the traditional Chinese culture of returning to one’s roots, preferred to die at home. However, families were fearful of managing end-of-life symptoms at home. When faced with these conflicts, professionals prioritized patient’s wishes and fostered consensus among families. As one nurse acknowledged, “The most important thing is to get a unity of opinion from the whole family. We have a family meeting to let them know what the patient really wants.” Moreover, discharging patients home required anticipatory symptom planning and clear communication, as one case manager noted: “We inform the family about possible risks...before letting them go back.” Postdischarge support via online tools extended care but also revealed hidden burdens: “Families get flustered....They’ll send messages at any time.”

#### Extending Care to Families After Patients’ Death

After a patient has passed away, the stable relationship established between professionals and family members does not disappear. Professionals continued to provide home visits and online grief counseling to help families gradually return to normal life. Some professionals would emphasize: “The level of grief of the families can reflect to some extent the quality of our services.” Likewise, the families were touched by the grief counseling provided by the professional team.

### The Components of Quality Home End-of-Life Care in China

Our findings reveal that the end-of-life care process is grounded in trust-building and relational development. The journey begins with assessing family readiness and service suitability, gradually earning acceptance for professional entry—“open the door.” Effective communication and practical support drive the continuity of the relationship throughout the service—“open the mouth.” Flexible home visits and tailored care allow patients and families to contribute proactively, fostering a mutually reciprocal relationship—“open the heart.” In the final phase, professionals support patients and families in navigating dying and death with dignity, honoring personal preferences and cultural values—“open the death.” These 4 elements define the components of quality home end-of-life care in China (Figure).

**Figure.**
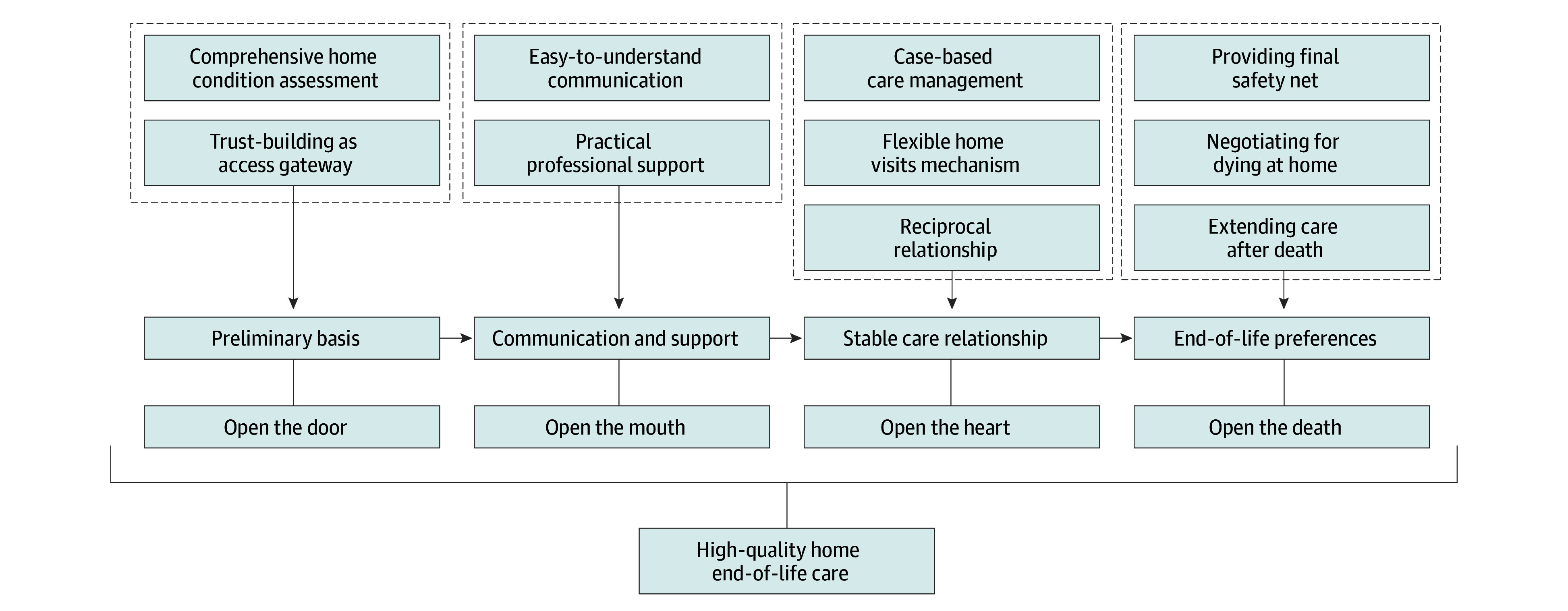
Components of Quality Home End-of-Life Care

## Discussion

This qualitative study, from the perspective of a multistakeholder and integrated approach using data from 4 different fields, provides a rich and nuanced understanding of the core elements of quality home end-of-life care in China. Through this study, we identified 4 interconnected core elements.

First, access to home end-of-life care in China was currently determined by informal criteria, heavily reliant on the presence of dedicated family caregivers and professional discretion. This prioritization may introduce service selection bias, especially in the context of the one-child policy and aging caregiver populations.^19,20^ Moreover, public unfamiliarity with home end-of-life care and natural privacy preservation contribute to initial wariness among some patients and families.^21^ Community workers can serve as the initial contact for home end-of-life care. A standardized access framework is needed to provide a more equitable and scalable solution for home end-of-life care access.

Second, effective communication is the cornerstone of quality home end-of-life care, including emotional and practical dimensions. Professionals adapted their language to patients’ cognitive and emotional readiness, enabling clearer understanding and greater comfort in discussing death-related topics.^22,23^ Beyond verbal support, practical assistance was essential. Professionals provided telemedicine, medication supply, and pain diaries for symptom control. However, strict narcotic regulations in China often limited drug accessibility, indicating the need for a fast-track medication safety and care access pathway for homebound patients.^24^ Furthermore, traditional Chinese massage was used for symptom relief, with families trained through professional demonstration and video instruction.^25^ Additional complementary therapies such as aromatherapy and reflexology could be explored with properly qualified professionals.^26^ Psychological interventions such as dignity therapy and life review have shown benefit but are neither routinely covered nor scaled up.^27^ Patients and families also expressed strong demand for end-of-life information, highlighting the need for integrated digital platforms to facilitate knowledge access and decision-making.^28^

Third, a dynamic of reciprocal trust between families and professional teams is cultivated over time through continuity of care and emotional resonance. Tools such as WeChat groups, personalized care files, and case-based team reflection meetings played crucial roles in tailoring services and ensuring accountability.^29^ Given the shortage of professionals in China, a flexible workforce model emerged (relatively fixed social workers and volunteers, flexible health care roles), but standardized volunteer training is still needed.^30^ This long-term and stable care support makes some patients and families also willing to share life experiences and engage in advocacy, aligning with the concept of reflexive hospice care, which emphasizes mutual emotional support and dignity-building at end-of-life.^31^

Lastly, fulfilling patients’ preferences regarding the place of death emerged as a defining feature of care quality. Rural patients often prefer dying at home, rooted in traditional beliefs of returning to one’s roots, whereas urban patients frequently choose institutional settings to avoid burdening families or triggering housing-related stigma. A trusting relationship built over time facilitates smooth patient referral from home to hospital, reflecting the importance of support continuity in home end-of-life care.^32^ Professionals actively mediated between patient wishes and family concerns. Although advance care planning remains underused in China,^33,34^ our findings suggest that professionals were already informally practicing its core principles. After death, grief counseling and follow-up visits helped families transition emotionally, reflecting a culturally grounded emphasis on relational continuity and family unity.^35,36^

These findings offer a foundation for translating key elements of quality home end-of-life care into meaningful practice and evaluation tools. Indicators such as timely and appropriate home access assessment, personalized communication, continuity of bereavement support, and fulfillment of the preferred place of death can be developed to guide service improvement. These indicators should be tested and adapted across varied care settings to ensure practical relevance. Future models should embrace a more holistic, family-centered approach—one that incorporates not only outcome measures but also the emotional and cultural dimensions of dying.

### Limitations

This study has limitations. Data collection was conducted in China’s capital region, Beijing, due to its relatively high concentration of home end-of-life care services. Broader regional sampling is required to enhance generalizability. Additionally, terminally ill patients’ declining health often precluded direct interviews, risking bias from reliance on observational data. Future research should expand geographic diversity and prioritize direct patient engagement to reduce bias.

## Conclusions

This study identifies 4 interconnected elements crucial for quality home end-of-life care in China: preliminary basis, communication and practical support, care relationships, and end-of-life preferences. These components form a trust-building dynamic between care providers and recipients, enriching the connotation of the framework for home end-of-life care. The findings provide practical guidance for advancing China’s home end-of-life care systems, urging policymakers to integrate these elements into quality assessment frameworks with corresponding resource allocation and policy support.

## Notes

Supplement 1. **eTable.** Interview Guideline

Supplement 2.
Data Sharing Statement
