# Supplementary material for: Important Elements of Quality Home End-of-Life Care in China
Source: JAMA Netw Open. 2025 Sep 10;8(9):e2531176. doi: 10.1001/jamanetworkopen.2025.31176 (PMC12423856; doi:10.1001/jamanetworkopen.2025.31176)
Supplement: Supplement 1. — eTable. Interview Guideline [file jamanetwopen-e2531176-s001.pdf]

Supplemental Online Content

Kan Y, Xiao Y, Li Z, et al. Important elements of quality home end-of-life care in China. *JAMA Netw Open*. 2025;8(9):e2531176. doi:10.1001/jamanetworkopen.2025.31176

eTable. Interview Guideline

This supplemental material has been provided by the authors to give readers additional information about their work.

© 2025 Kan Y et al. *JAMA Network Open*.

eTable. Interview Guideline

Participants	Interview guideline
**Professionals**	①Could you briefly provide an overview of the home end-of-life care services within our organization?<br>②What do you focus on in providing home end-of-life care services to terminally ill patients and families?<br>③Can you share one or two cases of a home end-of-life care service that you found to be more satisfying (successful/high quality)? What are some of the reasons or most important factors?<br>④What do you think are the fundamental elements of effective home end-of-life care? What are your expectations for quality development in home end-of-life care?<br>⑤Are there currently any activities related to quality evaluation of home end-of-life care in the organization? If so, can you briefly describe the process and effects of practice.
Patients and families	**The core question is:** How do you feel about the services (home end-of-life care) we provide ? What did you feel was good (high quality) about what we helped you do?<br>Specifics around the core question:<br>① Situation: What kind of difficulties did you encounter at that time?<br>② Action: What measures did we do for you?<br>③Results: After we did this for you, what impact did it have on you? What aspects do you think need to be further improved?

© 2025 Kan Y et al. *JAMA Network Open*.
