# Supplementary material for: Important Elements of Quality Home End-of-Life Care in China
Source: JAMA Netw Open. 2025 Sep 10;8(9):e2531176. doi: 10.1001/jamanetworkopen.2025.31176 (PMC12423856; doi:10.1001/jamanetworkopen.2025.31176)
Supplement: Supplement 2. — Data Sharing Statement [file jamanetwopen-e2531176-s002.pdf]

Data Sharing Statement

Kan. Important Elements of Quality Home End-of-Life Care in China. *JAMA Netw Open*. Published September 10, 2025. doi:10.1001/jamanetworkopen.2025.31176

Data
Data available: No
